# Usutu virus NS4A suppresses the host interferon response by disrupting MAVS signaling

**DOI:** 10.1016/j.virusres.2024.199431

**Published:** 2024-07-09

**Authors:** Tessa Nelemans, Ali Tas, Marjolein Kikkert, Martijn J. van Hemert

**Affiliations:** Molecular Virology Laboratory, Leiden University Center for Infectious Diseases (LUCID), Leiden University Medical Center, Leiden, The Netherlands

**Keywords:** Usutu virus/flavivirus/NS4A/interferon suppression/MAVS

## Abstract

Usutu virus (USUV) is an emerging flavivirus that can infect birds and mammals. In humans, in severe cases, it may cause neuroinvasive disease. The innate immune system, and in particular the interferon response, functions as the important first line of defense against invading pathogens such as USUV. Many, if not all, viruses have developed mechanisms to suppress and/or evade the interferon response in order to facilitate their replication. The ability of USUV to antagonize the interferon response has so far remained largely unexplored. Using dual-luciferase reporter assays we observed that multiple of the USUV nonstructural (NS) proteins were involved in suppressing IFN-β production and signaling. In particular NS4A was very effective at suppressing IFN-β production. We found that NS4A interacted with the mitochondrial antiviral signaling protein (MAVS) and thereby blocked its interaction with melanoma differentiation-associated protein 5 (MDA5), resulting in reduced IFN-β production. The TM1 domain of NS4A was found to be essential for binding to MAVS. By screening a panel of flavivirus NS4A proteins we found that the interaction of NS4A with MAVS is conserved among flaviviruses. The increased understanding of the role of NS4A in flavivirus immune evasion could aid the development of vaccines and therapeutic strategies.

## Highlights

• USUV nonstructural proteins including NS4A suppress interferon production.
• NS4A inhibits interferon production by counteracting MAVS signaling.
• NS4A blocks the interaction between MDA5 and MAVS.
• NS4A proteins from various flaviviruses suppress MAVS signaling.

## 1 Introduction

Over the past few decades many arboviruses have (re-)emerged, which has posed a substantial threat to public health (Pierson and Diamond, 2020). In Europe this includes Usutu virus (USUV), a positive-strand RNA virus belonging to the Flaviviridae family and closely related to West Nile virus (WNV). USUV can infect a large variety of hosts ranging from birds to mammals, including humans. USUV outbreaks are mainly associated with mortality in birds, and especially mass deaths among black birds, while human infections are mainly asymptomatic (Roesch et al., 2019). However, in rare cases the virus can cause severe neurological symptoms in humans, similar to the disease caused by WNV (Zannoli and Sambri, 2019). These severe USUV cases have been reported in Europe (Angeloni et al., 2023; Cavrini et al., 2009; Nagy et al., 2019; Santini et al., 2015; Simonin et al., 2018). With climate and environmental changes affecting the prevalence and distribution of mosquito vectors and the exposure of mammalian host populations to viruses (Medlock and Leach, 2015; Whitehorn and Yacoub, 2019), (emerging) arboviruses are expected to increasingly form a threat to human- and animal health.

**Fig. 1:**
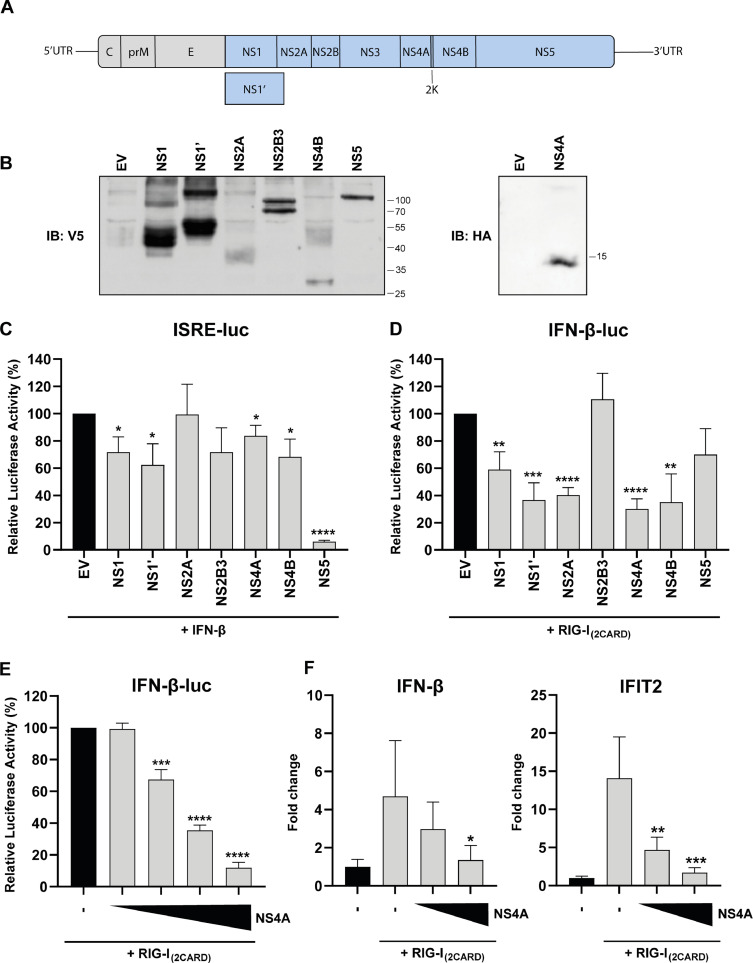
Multiple USUV nonstructural proteins inhibit IFN-β production and signaling. (A) Schematic overview of the USUV genome. The single open reading frames encodes a polyprotein that is proteolytically processed by viral and host proteases into structural proteins (depicted in grey) and non-structural proteins (depicted in blue). (B) Western blot showing expression of the USUV nonstructural proteins in 293T cells. (C) 293T cells were transfected with a combination of plasmids encoding 1) the ISRE-luc reporter, 2) *Renilla* luciferase as control for transfection efficiency, and 3) an USUV nonstructural protein. 16 hpt the cells were treated with 100 U/mL IFN-β and 8 h later luciferase activity was measured to quantify ISRE reporter activation. Means ± standard deviation of three independent experiments are shown. (D) 293T cells were transfected with a combination of plasmids encoding 1) the IFN-β-luc reporter, 2) *Renilla* luciferase as control for transfection efficiency, 3) RIG-I_(2CARD)_ and 4) an USUV nonstructural protein. At 16 hpt luciferase activity was measured. Means ± standard deviation of three independent experiments are shown. (E) 293T cells were transfected with the IFN-β-luc reporter, the *Renilla* luciferase control plasmid, the RIG-I_(2CARD)_ plasmid and increasing amounts of the USUV NS4A expression plasmid (125/250/500/1000 ng). At 16 hpt luciferase activity was measured. Means ± standard deviation of three independent experiments are shown. (F) 293T cells were transfected with the RIG-I_(2CARD)_ expression plasmid and 500 or 1000 ng of the NS4A expression plasmid. At 24 hpt cells were harvested and total RNA was isolated and the changes in IFN-β and IFIT2 mRNA levels were quantified by RT-qPCR. Means ± standard deviation of two independent experiments are shown. (C-F) Statistical significance relative to cells transfected with the empty vector (EV) is shown (* *p* < 0.05, ** *p* < 0.01 *** *p* < 0.001, **** *p* < 0.0001).

The USUV genome encodes one large polyprotein that is cleaved into three structural (C, prM, and E) and seven nonstructural (NS1, NS2A, NS2B, NS3, NS4A, NS4B, and NS5) proteins (Fig. 1A). NS5 contains the RNA dependent RNA polymerase (RdRp) necessary for replication of the viral genome, and additionally has methyltransferase (MTase) activity required for RNA capping. NS3 contains helicase, NTPase and protease activity, while NS2B functions as a co-factor for NS3 proteolytic activity. The other nonstructural proteins are thought to be involved in the formation of the viral replication complex. In addition, NS4A is a transmembrane protein that can induce membrane curvature (van den Elsen et al., 2021). Besides their essential role in the replication of the viral genome, these proteins also possess many other functions, including their important role in innate immune evasion (Chen et al., 2017). Due to a slippery sequence and a pseudoknot structure in the NS2A gene, in some cases a −1 ribosomal frameshift occurs during translation. This frameshift results in the production of an alternative form of NS1 named NS1’, which is an extended version of NS1 with 52 additional amino acids at its C-terminus (Melian et al., 2010). NS1’ plays a role in the pathogenesis of flaviviruses and has been shown to modulate the immune response of the infected host (Zhou et al., 2018a).

The host interferon (IFN) response is the first line of defense against invading viruses such as USUV (Stetson and Medzhitov, 2006). In response to viral infections cells can produce type I and type III IFNs (IFN-α/β and IFN-λ, respectively). These secreted IFNs regulate the expression of hundreds of genes, the so-called interferon-stimulated genes (ISGs), in order to promote an anti-viral state in the infected as well as in neighboring cells (Schoggins and Rice, 2011). Production of interferons is initiated after detection of viral replication intermediates, like double-stranded RNA or 5′-triphosphorylated RNA, by pattern recognition receptors (PRRs), such as retinoic acid-inducible gene I (RIG-I) and melanoma differentiation-associated protein 5 (MDA5) (Akira et al., 2006). After sensing these viral replication intermediates, RIG-I and MDA5 will translocate to mitochondria, peroxisomes, and mitochondria-associated membranes (MAMs) to activate the adaptor protein mitochondrial antiviral signaling (MAVS) via an interaction between their caspase recruitment domain (CARD) domains (Vazquez and Horner, 2015). Besides the CARD domain, MAVS also contains a C-terminal transmembrane domain, which anchors MAVS to e.g. the mitochondria, and it contains several TRAF-interacting motifs (TIMs), which enables the recruitment of the tumor necrosis factor receptor-related factor (TRAF) family members (TRAF2/3/5/6) after MAVS is activated (Ren et al., 2020; Vazquez and Horner, 2015). The recruited TRAF proteins will then promote the activation of the TANK-binding kinase 1 (TBK1) and inducible IκB kinase ε (IKKε). These kinases will phosphorylate the transcription factor interferon regulation factor 3 (IRF3), which upon activation and translocation to the nucleus will initiate the production of IFN-α/β and/or IFN-λ (Nelemans and Kikkert, 2019; Zhou et al., 2018b). Binding of IFNs to its cognate receptor leads to activation of Janus kinase 1 (JAK1) and tyrosine kinase 2 (TYK2). These kinases phosphorylate signal transducer and activator of transcription proteins (STAT1 and STAT2), which together with IRF9 form the IFN-stimulated gene factor 3 (ISGF3) complex. This complex will then trigger the transcription of the ISGs (Ivashkiv and Donlin, 2014; McNab et al., 2015).

To enable successful replication within the host, many, if not all, viruses have evolved diverse ways to counteract the IFN response. Numerous flaviviruses, including WNV, are well-known to inhibit type I IFN responses (Gack and Diamond, 2016; Zoladek and Nisole, 2023). Several of the WNV nonstructural proteins have been shown to target the IFN response, and have diverse ways to antagonize this response ranging from evading sensing, directly inhibiting components of the IFN pathway to inhibiting ISG function (Daffis et al., 2010; Laurent-Rolle et al., 2010; Liu et al., 2004, 2005; Zhang et al., 2017). Much less is known about how USUV interacts with the host's innate immune responses to evade their antiviral functions. IFN treatment of USUV-infected cells was much less effective post infection than when the cells were pretreated with IFN (Scagnolari et al., 2013), suggesting that USUV possesses the ability to counteract the IFN response during replication. However, it remains unknown which USUV proteins (or other molecules) are responsible for antagonizing the interferon response and through which mechanism(s).

In this study, we screened the nonstructural proteins of USUV for their ability to antagonize IFN-β production and signaling. We identified USUV NS4A as a strong antagonist of IFN production, and discovered that USUV NS4A interacts with MAVS. We mapped the responsible interacting domains in both proteins, and found that NS4A binding to MAVS blocks the interaction of MAVS with other signaling proteins from the IFN pathway. Testing NS4A proteins of a wide variety of other flaviviruses, demonstrated that the interaction (and IFN antagonism) is conserved among these flaviviruses, pointing to a common mechanism employed by these viruses to prevent a MAVS-dependent interferon response.

## 2 Materials and methods

### 2.1 Cell lines and viruses

A549 (Collection Medical Microbiology, LUMC), BHK-21 J (Lindenbach and Rice, 1997), Vero CCL-81 (Collection Medical Microbiology, LUMC), H1299 (a kind gift from Marc Vooijs, Maastricht University, the Netherlands), HEK293 (a kind gift from Annemarthe van der Veen, Leiden University Medical Center, the Netherlands) and 293T (ATCC CRL-3216) cells were grown at 37 °C in a 10 % CO_2_ (HEK293) or 5 % CO_2_ (all others) incubator. A549, H1299 and Vero CCL-81 cells were maintained in Dulbecco's modified Eagle's medium (DMEM, Gibco) supplemented with 8 % fetal calf serum (FCS, Capricorn Scientific), and 50 units/mL of streptomycin/penicillin (Merck). HEK293 and 293T cells were grown in DMEM supplemented with 10 % FCS, 2 mM l-glutamine (Sigma), and 50 units/mL of streptomycin/penicillin. BHK-21 J cells were cultured in Glasgow's MEM (GMEM, Gibco) supplemented with 8 % FCS, 10 % tryptose phosphate broth (Gibco), 10 mM HEPES (Lonza), and 50 units/mL of streptomycin/penicillin.

The USUV isolate used in this study was the Netherlands 2016 strain (lineage Africa 3, GenBank: MH891847) (Rijks et al., 2016). Virus stocks were grown in Vero CCL-81 cells until passage 4, and titrated in BHK-21 J cells by plaque assay essentially as described before (Kovacikova et al., 2020). A549 cells were then used to assess the immune response after USUV infection. USUV infections in A549 cells were performed at a multiplicity of infection (MOI) of 1 in low serum medium (DMEM + 2 % FCS). Sendai virus (SeV, Cantell strain) was purchased from Charles River Laboratories. SeV infection in A549 cells was performed using 100 haemagglutinin units (HAU)/mL in low serum medium. As a positive control, to induce the innate immune response, A549 cells were transfected with 1 μg/mL poly(I:C) (HMW, Invivogen) using lipofectamine 2000, following the manufacturers instructions. All the other transfections were performed in the 293T and HEK293, because of the high transfection efficiency we could accomplish in these cells.

### 2.2 Plasmids

The sequences encoding the USUV nonstructural proteins were amplified by PCR using a full-length cDNA clone (based on Netherlands 2016 strain, GenBank: MH891847) as template, and they were cloned into the mammalian expression vector pCAGGS (Addgene) by standard restriction enzyme based methods. USUV NS4A was fused to a N-terminal HA tag, NS2B3 was expressed with a N-terminal HA-tag and a C-terminal V5 tag, while the other USUV proteins were expressed with a N-terminal V5 tag (NS5) or C-terminal V5 tag (NS1, NS2A, NS4B). HA-tagged WNV and ZIKV NS4A plasmids were constructed as described above using the WNV Nea Santa-Greece-2010 full-length cDNA clone (GenBank: HQ537483) and the ZIKV SL1602 full-length cDNA clone (GenBank: KY348640) as template, respectively. DENV2 (GenBank: NC_001474), JEV (GenBank: NC_001437), YFV (GenBank: NC_002031), and TBEV (GenBank: NC_001672) NS4A sequences were ordered as synthetic DNA fragments (Twist Bioscience and GeneArt ThermoFisher) and subsequently cloned into the pCAGGS expression vector. A MUSCLE alignment of the NS4A sequences was performed using Geneious software (version 10.2.6). The USUV NS1’ expression construct was built by assembling two overlapping fragments into the pCAGGS vector using the NEBuilder® HiFi DNA Assembly Cloning Kit. The first fragment consisted of the NS1 sequence with an additional nucleotide at the 3′ end to mimic the −1 ribosomal frameshift, and the second fragment contained the sequence after the −1 ribosomal frameshift.

The following plasmids have been described previously: pLuc-IFN-β (Fitzgerald et al., 2003), pEF-RIG-I_(2CARD)_ (Gack et al., 2007), pcDNA3.1-FLAG-MAVS (Feng et al., 2014), and pEGFP-C1-IRF3_(5D)_ (Lin et al., 1998). The ISG54-ISRE (IFN-sensitive responsive element) reporter plasmid was a kind gift from Dr. Gijs Versteeg (Max Perutz Labs, Vienna, Austria). pEF-MDA5 was a kind gift from Dr. F. van Kuppeveld (Faculty of Veterinary Medicine, Utrecht, the Netherlands). pCAGGS-MAVS was a kind gift from Dr. N. Frias-Staheli (Icahn School of Medicine at Mount Sinai, New York, USA). pcDNA3.1-TBK1 was a kind gift from Dr. J. Hiscott (Istituto Pasteur Italia - Fondazione Cenci Bolognetti, Rome, Italy). IKKε-FLAG was a kind gift from Dr. M. Ressing (LUMC, Leiden, the Netherlands). pRL-TK which expresses *Renilla* luciferase was purchased from Promega.

Plasmids expressing the different MAVS domains were generated by amplifying specific regions of the full-length pCAGGS-MAVS construct using PCR, followed by restriction enzyme cloning into pCAGGS or pcDNA3.1. EV-pCAGGS-GFP was created by amplifying the eGFP region from pCAGGS-IRES-GFP (Treffers et al., 2023), followed by restriction enzyme cloning into the pCAGGS vector. GFP-tagged truncated forms of USUV NS4A were amplified using the full-length construct as a template and then cloned in frame with GFP into the pCAGGS-GFP vector. The USUV NS4A truncations were based on an sequence alignment with the predicted domains of DENV NS4A (Miller et al., 2007).

All primers used for construction of plasmids are listed in table S1. All constructs were verified by Sanger sequencing.

### 2.3 Dual-luciferase reporter assay

Using the transfection reagent polyethylenimine (PEI 25 K, Polysciences), 293T cells in 24-wells plates (1,5 × 10^5^ cells per well) were co-transfected with a reporter plasmid IFN-β-Luc (50 ng), an internal control plasmid expressing *Renilla* luciferase (5 ng), innate immune response inducing plasmids (25–50 ng) expressing RIG-I_(2CARD),_ MDA5, MAVS, TBK1, IKKε, or IRF3_(5D)_ and plasmids expressing the viral proteins (500 ng). Empty vector DNA was added to the transfection mixes to ensure that the total amount of DNA transfected was equal for all combinations of plasmids used. 16 h post transfection (hpt) cells were lysed and luciferase activity was determined using a Dual-Luciferase Reporter Assay System (Promega) and an EnVision multiplate reader (PerkinElmer). Transfection of the ISRE-Luc reporter was performed as above, except that no plasmids were used to induce the innate immune response. Instead, cells were treated with 500 U/mL IFN-β (PBL Assay Science) at 16 hpt, followed by lysis and analysis 8 h later. The assays were repeated independently at least three times, and each separate experiment contained quadruplicate samples. Relative luciferase activity was calculated by normalizing Firefly luciferase activity to *Renilla* luciferase activity, followed by determining the fold change compared to the empty vector control. An unpaired two-tailed Student's *t*-test was performed to check for statistical significance.

### 2.4 RNA isolation and real-time qPCR

After removal of the medium, infected or transfected cells were lysed in a buffer containing 3 M guanidine-thiocyanate, 2 % N-lauroylsarcosine, 50 mM Tris–HCl (pH 7.6), and 20 mM EDTA. RNA was isolated from the cell lysates using the Bio-on-Magnetic-Beads (BOMB) method (Oberacker et al., 2019). The RNA was then reverse-transcribed into cDNA using the RevertAid H Minus reverse transcriptase (Thermo Scientific) and random hexamers. To measure the host interferon response a real-time quantitative PCR was then performed with iQ SYBR green Supermix (Biorad). The samples were run in a CFX384 Touch real-time PCR detection system (Bio-Rad) using the following program: 3 min at 95 °C and 30 s at 60 °C followed by 40 cycles of 10 s at 95 °C, 10 s at 60 °C and 30 s at 72 °C. Gene expression was quantified by the standard curve method, and then normalized to expression of RPL13a. The fold change compared to the control samples was calculated and then plotted in GraphPad Prism (version 9). An unpaired two-tailed Student's *t*-test was performed to check for statistical significance. The primers used for RT-qPCR are listed in table S2.

### 2.5 Western blotting

To obtain protein lysates, cells were lysed in 2x Laemmli sample buffer (LSB). Proteins were separated by sodium dodecyl sulfate-polyacrylamide gel electrophoresis (SDS-PAGE) and then transferred onto a nitrocellulose membrane by semi-dry blotting using a Trans-blot Turbo system (Bio-rad). Membranes were blocked in 5 % dried milk powder in PBS with 0.05 % Tween-20 (PBST) for 1 hour, followed by overnight incubation with the primary antibody at 4 °C. The following primary antibodies were used: mouse anti-HA (clone HA.C5, Abcam), mouse anti-V5 (clone 2F11F7, Thermo Fisher/Invitrogen), and rabbit anti-GAPDH (clone 14C10, Cell Signaling Technology). After incubation with a horseradish peroxidase-conjugated secondary antibody, the blots were visualized with Amersham ECL Plus detection reagent (GE Healthcare).

For immunoprecipitation samples the western blotting protocol was adapted as follows: PVDF membranes (Merck Millipore) were blocked in 1 % casein in PBST for 1 hour, followed by overnight incubation with the primary antibody at 4 °C. The following primary antibodies were used: mouse anti-HA (HA.C5, Abcam), rabbit anti-FLAG (#2368, Cell Signaling Technology), rabbit anti-GFP (042,150, Leiden (van Kasteren et al., 2012)), rabbit anti-GAPDH (clone 14C10, Cell Signaling Technology), mouse anti-Vinculin (clone 2B5A7, ProteinTech), rabbit anti-Lamin B1 (polyclonal 12987-1-AP, Proteintech) rabbit anti-MAVS (clone D5A9E, Cell Signaling Technology), rabbit anti-MAVS (polyclonal #3993, Cell Signaling Technology) and mouse anti-MAVS (clone E-3, Santa Cruz). After incubation with a biotin-conjugated secondary antibody and a Cy3-conjugated tertiary antibody, the blots were visualized using an Alliance Q9 Advanced imaging system (Uvitec).

### 2.6 Immunoprecipitation assay

293T cells (in 10 cm dishes or 6-wells plate) were transfected with the indicated NS4A and MAVS constructs using PEI 25 K. HEK293 cells (in 10 cm dishes) were transfected with plasmids expressing MDA5 or TRAF3, MAVS and USUV NS4A using lipofectamine 3000. At 24 hpt cells were washed once with PBS and harvested on ice in IP lysis buffer (20 mM Tris–HCl pH 7.4, 135 mM NaCl, 1 % Triton X-100, 10 % glycerol supplemented with cOmplete protease inhibitors (Roche)). Protein lysates were cleared by centrifugation (1.000 x g, 15 min, 4 °C) and a fraction of the whole cell lysate was saved for downstream analysis. The rest of the lysate was used for the immunoprecipitation using Pierce™ DYKDDDDK Magnetic Agarose (Thermo Scientific) or Pierce Anti-HA Magnetic Beads (Thermo Scientific), following the manufacturer's instructions. In short, pre-washed magnetic agarose (50 μl) was added to the lysates and incubated for 4 h or overnight at 4 °C with gentle agitation. Next, the beads were washed three times with PBS containing 0.3 M NaCl and 0.1 % Triton X-100, and once with ultrapure water. The proteins were eluted from the beads using 2x LSB. Proteins were then separated by SDS-PAGE and detected by western blotting.

### 2.7 Immunofluorescence assay

H1299 and 293T cells were grown on glass coverslips and transfected using lipofectamine 3000 or PEI 25 K, respectively, according to the manufacturer's protocol. At 24 hpt, cells were fixed in 3 % paraformaldehyde in PBS. Subsequently, cells were washed twice with PBS containing 10 mM glycine and permeabilized with 0.1 % Triton X-100 in PBS for 10 min, followed by incubation with the primary antibody for 1 h. Primary mouse anti-PDI (clone 1D3, Enzo Life Sciences), rabbit anti-TOMM20 (ab78547, Abcam), anti-ACSL4 (clone F-4, Santa Cruz), mouse anti-MAVS (clone E-3, Santa Cruz), and rabbit anti-MAVS (clone D5A9E, Cell Signaling Technology) antibodies were diluted in PBS with 5 % FCS. Primary mouse anti-HA (clone 12CA5, Roche), and rabbit anti-IRF3 (clone D6I4C, Cell Signaling Technology) antibodies were diluted in PBS with 1 % BSA. The coverslips were washed with PBS and incubated in the dark with the following secondary antibodies: donkey anti-mouse conjugated to Cy3 (Jackson ImmunoResearch) and donkey anti-rabbit conjugated to Alexa 647 (Jackson ImmunoResearch), or goat anti-mouse conjugated to Alexa 488 (Thermo Fisher) and donkey anti-rabbit conjugated to Cy3 (Jackson ImmunoResearch). Nuclei were stained with Hoechst 33258 (Thermo Fisher). The coverslips were mounted on slides with ProLong Gold Antifade reagent (Thermo Fisher), and images were taken with a Leica SP8 confocal microscope or DM6B fluorescence microscope using LASX software.

## 3 Results

### 3.1 USUV nonstructural proteins inhibit IFN-β production and signaling

To assess the effect of USUV on the IFN response, we measured IFN-β, interferon-induced protein with tetratricopeptide repeats 2 (IFIT2) and ISG15 mRNA levels in USUV-infected A549 cells and compared them to Sendai virus (SeV) infected cells. SeV is known to induce a strong and rapid IFN response and as expected, we observed a rapid increase in IFN-β, IFIT2 and ISG15 mRNA levels after SeV infection. This response was already measurable as early as 4 h after SeV infection, while an increase in SeV genome copies was only detected at 6 hpi (Fig. S1A-D). USUV induced the expression of these genes to even higher levels, but their upregulation was delayed compared to SeV. USUV replication was already evident at 12 hpi, while an increase in IFN-β and ISG levels was only observed after 18 h (Fig. S1A-D). This suggests that USUV possesses the ability to suppress the early induction of the innate immune response. This is further supported by the observation that already at 6 hpt stimulation of A549 cells with poly(I:C) resulted in a large increase of IFN-β, IFIT2 and ISG15 mRNA levels (Fig. S1E), showing that the cells are capable of a rapid and strong IFN response.

To determine which USUV proteins are responsible for suppressing the innate immune response, we constructed plasmids to express the individual nonstructural proteins of USUV (Fig. 1A). These proteins were tested in dual-luciferase assays to analyze their effect on either IFN-β production or IFN signaling (by measuring the ISG54-ISRE promotor activity). In these assays, 293T cells were transfected with an IFN-β or ISG54-ISRE reporter plasmid, *Renilla* luciferase for normalization purposes, and one of the plasmids coding for the different USUV proteins fused to a V5 or HA tag. Western blot analysis confirmed that cells transfected with the USUV expression constructs expressed proteins of the expected sizes (Fig. 1B). To induce IFN-β promoter activity, cells were co-transfected with constitutively active RIG-I_(2CARD)_, while the ISG54-ISRE reporter was activated by treating the cells with IFN-β. ISG54-ISRE promoter activity was inhibited most effectively by USUV NS5 (Fig. 1C). This was expected since STAT antagonism by NS5 is well-conserved among flaviviruses (Ashour et al., 2009; Grant et al., 2016; Laurent-Rolle et al., 2010). Luciferase expression of the IFN-β reporter was significantly inhibited by all of the USUV nonstructural proteins, except for NS2B3 and NS5 (Fig. 1D). From the proteins tested, USUV NS4A suppressed the IFN-β promoter activity to the largest extent, as they reduced luciferase levels to ∼30 % of the activity found in cells co-transfected with the empty vector (EV) (Fig. 1D). We decided to further investigate the immune evasion activity of USUV NS4A and could show a dose-dependent inhibitory effect of USUV NS4A in the luciferase assays (Fig. 1E). The inhibitory effect was confirmed in an independent system, as overexpression of USUV NS4A significantly reduced IFN-β and IFIT2 mRNA levels in 293T cells stimulated with RIG-I_(2CARD)_ (Fig. 1F).

### 3.2 USUV NS4A inhibits IFN production upstream of IRF3

**Fig. 2:**
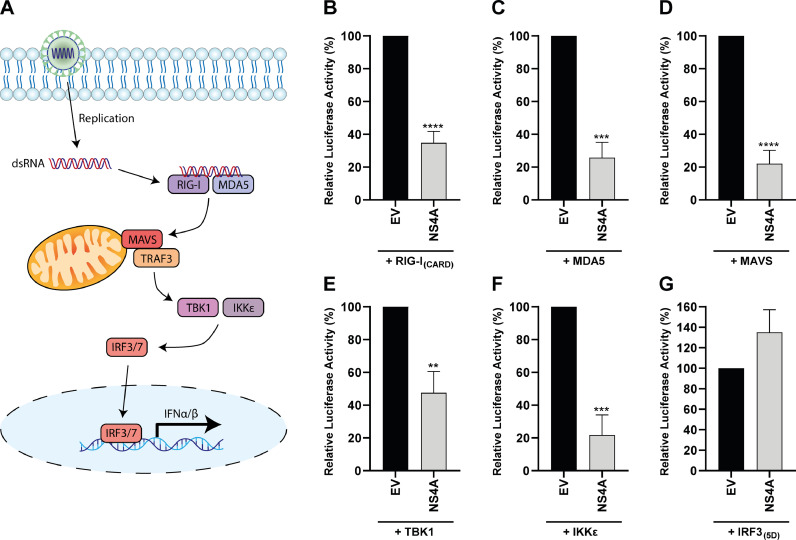
USUV NS4A inhibits IFN-β production by targeting the pathway upstream of IRF3. (A) Schematic overview of the RIG-I/MDA5 mediated IFN-β production pathway. (B-G) IFN-β reporter assays using innate immune response inducers at different levels in the signalling cascade as depicted in (A). 293T cells were transfected with the IFN-β-luc reporter plasmid, the *Renilla* luciferase expression plasmid as control for transfection efficiency, the USUV NS4A expression plasmid and plasmids encoding either RIG-I_(2CARD)_ (B), MDA5 (C), MAVS (D), TBK1 (E), IKKε (F), or IRF3_5D_ (G). At 16 hpt luciferase activity was measured. Means ± standard deviation of three independent experiments are shown. Statistical significance relative to cells transfected with the empty vector (EV) is shown (** *p* < 0.01 *** *p* < 0.001, **** *p* < 0.0001).

To determine which step(s) in the IFN-β production pathway is targeted by USUV NS4A, we performed additional luciferase assays with the IFN-β reporter plasmid. We overexpressed different components of this signaling pathway to activate the signaling cascade at different levels of the pathway (Fig 2A), in combination with NS4A co-expression. USUV NS4A was no longer able to suppress activation of the IFN-β reporter when the pathway was activated by overexpression of IRF3_(5D)_, a constitutively active form of IRF3 (Fig. 2G). However, USUV NS4A was able to suppress IFN-β promoter activity when the pathway was activated more upstream (Fig. 2B-G). This suggests that USUV NS4A acts on proteins upstream of IRF3.

### 3.3 USUV NS4A interacts with MAVS and localizes at the mitochondria-associated membranes

**Fig. 3:**
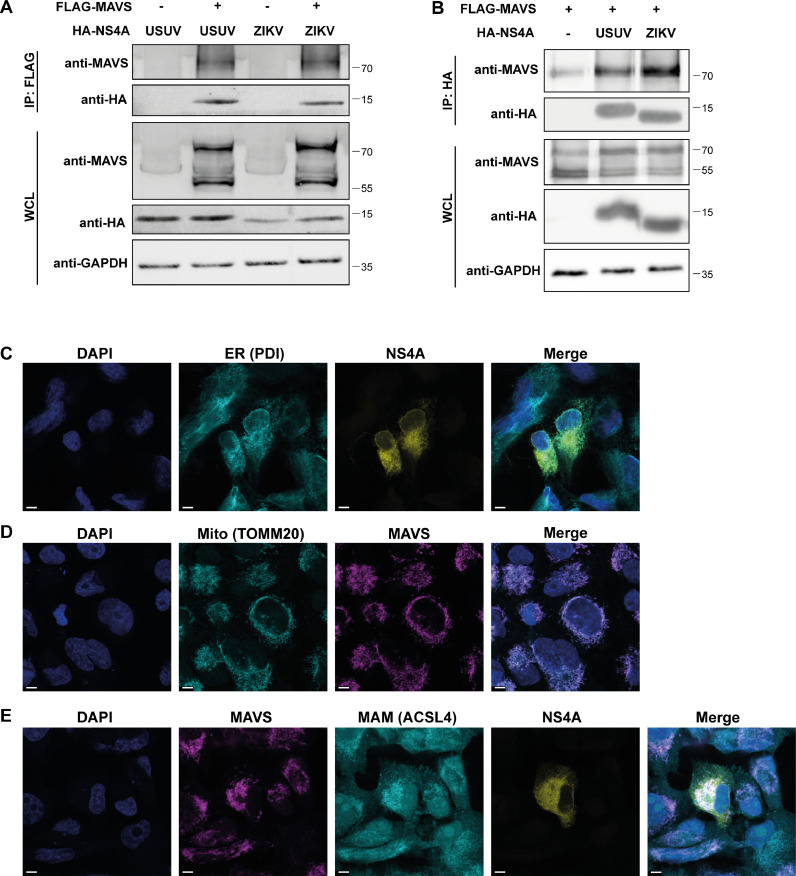
USUV NS4A interacts with MAVS and co-localizes at MAMs. (A-B) 293T cells were co-transfected with expression plasmids for HA-tagged USUV or ZIKV NS4A and FLAG-tagged MAVS. At 24 hpt lysates were collected and immunoprecipitated with a FLAG antibody (A) or HA antibody (B) and the whole cell lysates and immunoprecipitates were analysed for the indicated proteins by western blot. (C-E) H1299 cells were transfected with a plasmid encoding GFP-tagged NS4A and at 24 hpt the cells were fixed and stained for the ER marker PDI (C), MAVS and mitochondrial marker TOMM20 (D), MAVS and MAM marker ACSL4 (E). Nuclei were visualized by Hoechst 33258. Overlap of the fluorescent signals in the merge channel can be seen as green (C), purple (D) or white (E). Scale bars in the bottom left corner represent 5 μm.

Since DENV and ZIKV NS4A were shown to interact with MAVS (He et al., 2016; Hu et al., 2019; Ma et al., 2018), we tested whether USUV NS4A also interacted with MAVS, as a potential mechanism to inhibit IFN production. To this end, we performed a co-immunoprecipitation assay using 293T cells transfected with HA-tagged USUV NS4A or ZIKV NS4A (as a positive control) and FLAG-tagged MAVS. Using a FLAG antibody to precipitate MAVS, we found that both USUV and ZIKV NS4A co-precipitated with MAVS, but not with the EV control (Fig. 3A). This showed that USUV NS4A similar to ZIKV NS4A can bind to MAVS. We confirmed the NS4A-MAVS interaction by pull-down of NS4A with an HA antibody and found co-immunoprecipitation of MAVS (Fig. 3B). NS4A is a transmembrane protein that localizes to the ER (Fig. 3C), while MAVS can be found on mitochondrial membranes (Fig. 3D) or peroxisomes (Vazquez and Horner, 2015). The ER contains a specialized part that forms contact sites with the mitochondria, the so-called mitochondria-associated membranes (MAMs) (Vance, 2014). We tested by immunofluorescence whether USUV NS4A localized to the MAMs to interact with MAVS. We stained for MAVS and the MAM-enriched marker ACSL4 (Lewin et al., 2001) in cells transfected with GFP-NS4A. Both NS4A and MAVS partially co-localized with ACSL4 (overlap of all three markers can be seen as white in the merge image in Fig. 3E), suggesting that both proteins were indeed present at the MAMs.

### 3.4 USUV NS4A interacts with the CARD and TM domains of MAVS and disrupts the interaction between MAVS and MDA5

**Fig. 4:**
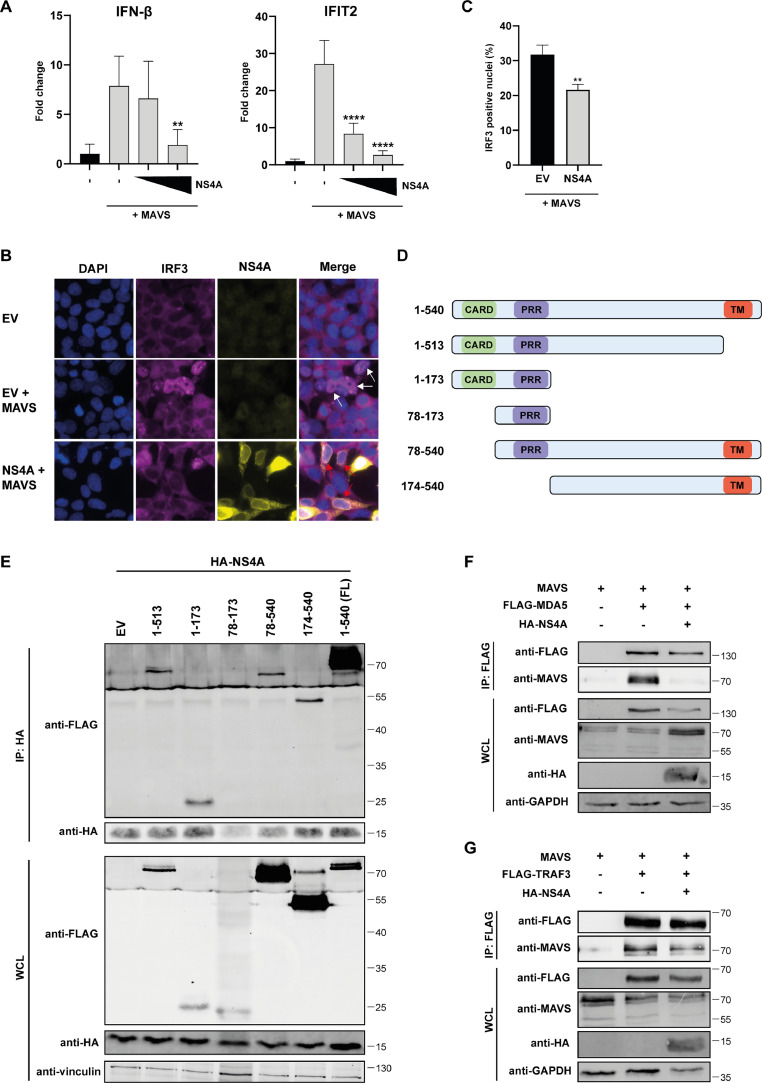
USUV NS4A interacts with the CARD and TM domains of MAVS and blocks the interaction of MAVS with MDA5 and TRAF3. (A) 293T cells were transfected with the MAVS expression plasmid and increasing amounts of the NS4A expression plasmid. At 24 hpt cells were harvested and total RNA was isolated to quantify the changes in IFN-β and IFIT2 mRNA levels by RT-qPCR. Means ± standard deviation of two independent experiments and statistical significance relative to empty vector-transfected cells is shown (** *p* < 0.01, **** *p* < 0.0001). (B-C) 293T cells were co-transfected with the MAVS expression plasmid and either the empty vector or NS4A expression plasmid. Cells were fixed 17 hpt and stained for IRF3 and HA (NS4A). Nuclei were visualized by Hoechst 33258. White arrows indicate cells with IRF3 in the nucleus. Red arrows indicate cells expressing NS4A without IRF3 translocation to the nucleus (B). The percentage of nuclei positive for IRF3 was quantified and plotted (C). Three different images were used for counting the number of cells positive for IRF3 translocation (>200 cells counted per image). ImageJ and QuPath (version 0.5.0) software was used to manually count the cells. Means ± standard deviation and statistical significance relative to the EV-transfection are shown (** *p* < 0.01). (D) Schematic overview of the different MAVS constructs used in the immunoprecipitation assay. (E) 293T cells were co-transfected with plasmids expressing HA-tagged USUV NS4A and different FLAG-tagged MAVS constructs. At 24 hpt lysates were collected and immunoprecipitated with a HA antibody. The whole cell lysates and immunoprecipitates were analysed for the indicated proteins by western blot analysis. (F-G) HEK293 cells were transfected with plasmids expressing HA-tagged NS4A, MAVS, and FLAG-tagged MDA5 (F), or FLAG-tagged TRAF3 (G). At 24 hpt lysates were collected and immunoprecipitated with a FLAG antibody. The whole cell lysates and immunoprecipitates were analysed for the indicated proteins by western blot.

To validate that NS4A can inhibit MAVS-induced signaling in an assay different from the luciferase assay, we studied the effect of NS4A on the mRNA levels of IFN-β and IFIT2 in cells stimulated by MAVS overexpression. When compared to the EV control, NS4A significantly decreased mRNA levels of both IFN-β and IFIT2 after stimulation of the cells (Fig. 4A). Next we determined whether MAVS-induced nuclear translocation of IRF3 could be inhibited by NS4A. IRF3 was not present in the nucleus when cells were not stimulated, but after overexpression of MAVS IRF3 translocation to the nucleus could be observed (Fig. 4B, middle panels). When NS4A was co-expressed with MAVS in the cells, IRF3 translocation was inhibited resulting in a reduced total number of cells showing IRF3 translocation (Fig. 4B, lower panels and Fig. 4C), further supporting the observation that NS4A inhibits MAVS-induced signaling.

Next, we investigated how the interaction of NS4A with MAVS might influence MAVS signaling. MAVS is activated when its N-terminal CARD domain interacts with the CARD domains of RIG-I or MDA5. Activated MAVS then recruits TRAF proteins through the TRAF-interacting motifs, which are located in the proline-rich region (PRR) and near the TM domain of MAVS. This interaction then leads to further signal transduction to induce the interferon response (Vazquez and Horner, 2015). We constructed plasmids expressing different parts of MAVS (Fig. 4D) to see which functional domains of MAVS are bound by USUV NS4A to potentially reduce MAVS signaling. We then performed co-immunoprecipitation assays with the different MAVS constructs and USUV NS4A. We observed that only the PRR construct did not co-precipitate with NS4A (Fig. 4E). This suggests that the CARD domain and TM part of MAVS can both interact with NS4A. However, the interaction of the truncated MAVS proteins with NS4A was clearly weaker than the interaction with the full-length MAVS, which might be caused by changes in folding and localization of the truncated proteins.

Next, we investigated whether binding of NS4A to MAVS can disrupt the complex formation of MAVS with the upstream factors such as MDA5 and/or downstream factors such as TRAF3. For technical reasons we used HEK293 for this experiment. HEK293 cells were transfected with untagged MAVS, HA-tagged NS4A and FLAG-tagged MDA5 or FLAG-tagged TRAF3 followed by a FLAG immunoprecipitation. We then checked by western blot analysis whether MAVS co-precipitated with MDA5 and TRAF3 and whether this was reduced in the presence of USUV NS4A. After the IP of MDA5, MAVS levels were indeed decreased in the presence of USUV NS4A, which suggests that binding of NS4A to MAVS reduces the interaction between MAVS and the upstream sensors (Fig. 4F). After the IP of TRAF3, we also observed reduced levels of MAVS in the presence of USUV NS4A, but this decrease was only minor (Fig 4G). This suggests that NS4A also possesses some ability to block the interaction of MAVS with downstream factors.

### 3.5 The transmembrane domains of NS4A are required for the interaction with MAVS

**Fig. 5:**
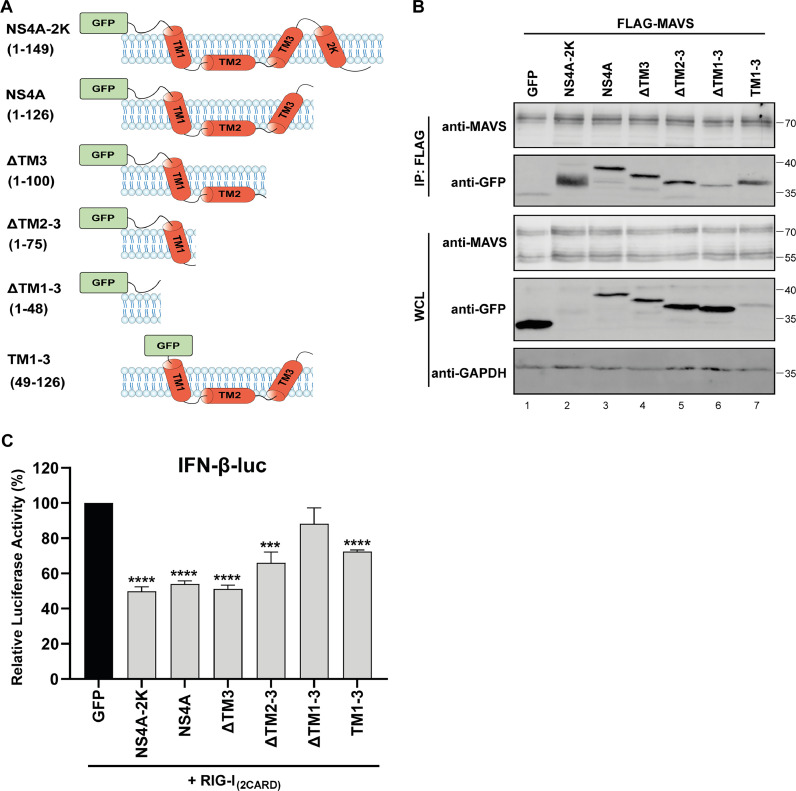
The transmembrane domains of NS4A are required for the inhibition of MAVS mediated signalling. (A) Schematic overview of the different NS4A constructs used in the immunoprecipitation assay. (B) 293T cells were co-transfected with expression plasmids for FLAG-tagged MAVS and the different GFP-tagged USUV NS4A constructs. At 24 hpt lysates were collected and immunoprecipitated with a FLAG antibody. The whole cell lysates and immunoprecipitates were analysed for the indicated proteins by western blot. (C) 293T cells were transfected with the IFN-β-luc reporter plasmid, *Renilla* luciferase plasmid, RIG-I_(2CARD)_ plasmid and the different NS4A deletion constructs. At 16 hpt luciferase activity was measured. Means ± standard deviation of three independent experiments are shown and statistical significance relative to GFP control-transfected cells is indicated (*** *p* < 0.001, **** *p* < 0.0001).

NS4A is a transmembrane protein that consist of a cytoplasmic tail and four transmembrane domains (TM1, TM2, TM3, and the 2K peptide). To map which of these domains are required for the interaction with MAVS, we created GFP-tagged truncated NS4A variants containing (combinations of) the various regions of the protein (Fig. 5A). These constructs were tested for their interaction with MAVS in co-immunoprecipitation assays, which included the EV expressing GFP as negative control. The TM2, TM3 and 2K peptide regions could be removed from NS4A without disturbing the interaction with MAVS (Fig 5B, lanes 3–5). Expression of the transmembrane regions (TM1–3) without the cytoplasmic tail was also sufficient to retain the interaction with MAVS (Fig. 5B, lane 7). The interaction with MAVS was only reduced when all TM domains including TM1 (ΔTM1–3) were deleted (Fig. 5B, lane 6). We then used the NS4A truncation constructs in the IFN-β reporter luciferase assays to determine which domains were required to suppress IFN-β promoter activity. In this assay it was clear that the ΔTM1–3 construct, which lacked all TM domains and lost its interaction with MAVS, was less able to suppress RIG-I_(2CARD)_-stimulated IFN-β reporter expression, compared to the other truncated NS4A proteins (Fig. 5C). However, the ΔTM2–3 and TM1–3 proteins also did not suppress the IFN-β promoter activity to the same extent as full-length NS4A. Altogether this suggests that TM1 might be sufficient to bind MAVS, but for the optimal inhibition of IFN-β production NS4A also needs its cytoplasmic tail and TM2 domain.

### 3.6 Interaction between NS4A and MAVS is conserved between flaviviruses

**Fig. 6:**
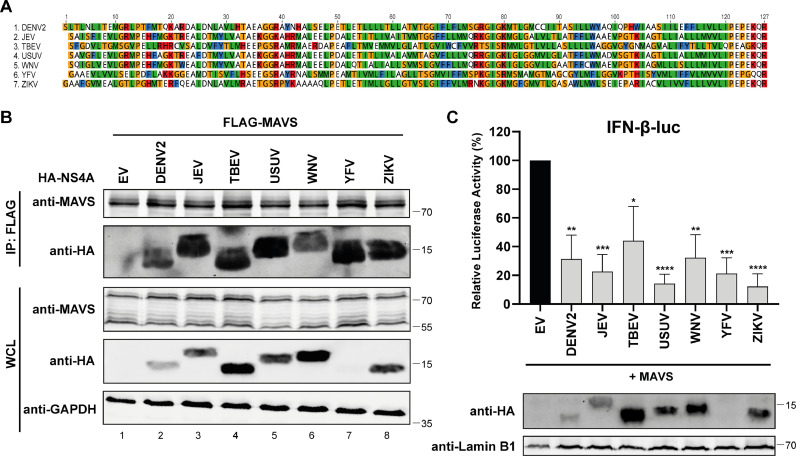
Flavivirus NS4A interacts with MAVS and inhibits MAVS-induced IFN-β responses. (A) Alignment of the flavivirus NS4A amino acid sequences (DENV2, JEV, TBEV, USUV, WNV, ZIKV, and YFV). Coloring of the amino acids is based on the ‘’clustal’’ setting: Orange = *G*, P, S, T; Green = *I*, L, M, V; Red = *H*, K, R; Blue = *F*, W, Y. (B) 293T cells were co-transfected with plasmids expressing FLAG-tagged MAVS and the different HA-tagged flavivirus NS4A constructs. At 24 hpt lysates were collected and immunoprecipitated with a FLAG antibody. The whole cell lysates and immunoprecipitates were analysed for the indicated proteins by western blot. (C) 293T cells were transfected with the IFN-β-luc reporter plasmid, *Renilla* luciferase plasmid, MAVS plasmid and the different flavivirus NS4A expression plasmids. At 16 hpt luciferase activity was measured. Means ± standard deviation of three independent experiments are shown and statistical significance relative to EV-transfected cells is indicated (* *p* < 0.05, ** *p* < 0.01 *** *p* < 0.001, **** *p* < 0.0001). To verify expression of the flavivirus NS4A proteins luciferase lysates were analysed by western blot.

We have shown here that USUV NS4A targets MAVS to inhibit IFN-β production, and earlier studies reported similar effects for DENV and ZIKV NS4A (He et al., 2016; Hu et al., 2019; Ma et al., 2018). This prompted us to the question whether the NS4A-MAVS interaction is also conserved for other flaviviruses. Besides DENV and ZIKV, we selected the most epidemiologically relevant flaviviruses (WNV, JEV, TBEV, YFV) and constructed expression plasmids for their NS4A proteins. An alignment of NS4A sequences (Fig. 6A) indicated that overall there is only a 10,2% identity at the amino acid level, but within the domains of NS4A the properties of the amino acids are highly similar between the different flaviviruses (amino acids with similar properties are indicated with the same color). This could mean that the functionality of NS4A is also conserved between the flaviviruses. We then co-transfected 293T cells with the expression constructs for FLAG-tagged MAVS and the HA-tagged NS4A proteins of the different flaviviruses and performed a co-immunoprecipitation assay in which we pulled down MAVS. All the tested NS4A proteins co-precipitated with MAVS (Fig. 6B). There were some differences in pulldown efficiency between the different flavivirus NS4A proteins. Strikingly, YFV NS4A levels were almost below the limit of detection in the whole cell lysate before the immunoprecipitation, while after the MAVS immunoprecipitation it was enriched to a level that gave rise to a strong band (Fig. 6B, lane 7). In contrast, after the immunoprecipitation the WNV NS4A protein was not as abundant compared to the NS4A protein levels of the other flaviviruses (Fig. 6B, lane 6), meaning that the pulldown efficiency of WNV NS4A was lower. Next, using luciferase assays in which we measured IFN-β promoter activity after stimulation by MAVS expression and co-expression of the flavivirus NS4A proteins, we found that each of the different NS4A proteins inhibited MAVS-induced IFN-β promoter activity (Fig. 6C). We again observed differences between the NS4A proteins of the different flaviviruses, as ZIKV NS4A caused the strongest reduction in luciferase activity, while TBEV NS4A was the least effective at reducing the activation of the IFN-β reporter. Altogether, despite the observed variation these results suggest that suppression of the MAVS-mediated interferon response is a conserved function of flavivirus NS4A proteins.

## 4 Discussion

Many viruses including the flaviviruses are known to counter the interferon response to facilitate their replication. Flaviviruses that have a decreased ability to suppress the IFN response were shown to be attenuated in vivo and differences in IFN antagonism can affect the virulence of viruses (Laurent-Rolle et al., 2010; Liu et al., 2004, 2006; Szretter et al., 2012; Xia et al., 2018). Thus, studying the mechanisms of innate immune evasion gives us important insights into the replication and pathogenesis of these viruses. Before this study, very little was known about innate immune evasion by USUV and none of its proteins had linked to this activity. Here we show that 6 of the USUV nonstructural proteins are capable (to varying extent) of suppressing either IFN-β production or signaling downstream of IFN in dual-luciferase reporter assays. Using these assays we showed that USUV NS5 efficiently inhibited the JAK/STAT signaling pathway, a property that is well-conserved among flaviviruses (Ashour et al., 2009; Best, 2017; Grant et al., 2016; Laurent-Rolle et al., 2010). Interestingly, the mechanism by which NS5 antagonizes JAK/STAT signaling varies between the different flaviviruses and the exact mechanism by which USUV NS5 suppresses this pathway remains to be determined.

According to our results, the IFN-β production pathway was targeted by several USUV proteins, i.e. NS1, NS1’, NS2A, NS4A, and NS4B, when they were overexpressed individually. This is in line with data obtained for other flaviviruses, which showed that these individual proteins can antagonize the IFN pathway (Dalrymple et al., 2015; He et al., 2016; Wu et al., 2017; Zhang et al., 2017; Zhou et al., 2018a). The strongest reduction in IFN-β promoter activity was observed after overexpression of USUV NS4A. Therefore, we continued to better characterize the innate immune evasion activity of USUV NS4A. We found that USUV NS4A interacted with MAVS and impaired MAVS-induced signaling, in line with earlier findings on DENV and ZIKV NS4A (He et al., 2016; Hu et al., 2019; Ma et al., 2018). USUV NS4A interacted specifically with the CARD and TM domains of MAVS and disrupted the interactions between MDA5 and MAVS, and to a lesser extent the interactions between TRAF3 and MAVS. This might indicate that the main mechanism of action of NS4A is interfering with the interaction of MAVS with upstream factors, although it also possesses some ability to block downstream signaling of MAVS. The CARD domain of MAVS is required for the interaction with the sensors MDA5 and RIG-I (Kawai et al., 2005), while it has been shown that the TM domain influences the oligomerization of MAVS and subsequently binding to TRAF3 (Tang and Wang, 2009). We therefore propose that USUV NS4A binding to the CARD domain of MAVS interferes with the interaction with MDA5, while its binding to the C-terminal part of MAVS might influence aggregation of MAVS and the interaction with the TRAF proteins. In this study we have only shown the effect of NS4A on the interactions between MDA5 and MAVS. However, it would be interesting to examine the effect on RIG-I signaling as well, since both RIG-I and MDA5 are thought to be essential for the recognition of flaviviruses (Errett et al., 2013; Loo et al., 2008).

Since NS4A is mainly localized to the ER, NS4A is most likely to encounter MAVS at the ER-mitochondria contact sites known as MAMs. The MAMs have been shown to play an important role in innate immune signaling and can be targeted by viruses to evade the antiviral response (Missiroli et al., 2018). For example, the protease NS3/4A from hepatitis C virus has been shown to specifically cleave MAVS located at the MAMs (Horner et al., 2011). Our data show that USUV NS4A can co-localize with MAVS at the MAMs, where it likely interferes with the interactions between MAVS and other innate immune factors, thereby suppressing IFN production.

Interestingly though, we noticed that TBK1- and IKKε-induced IFN-β production was also inhibited by NS4A. This suggests that USUV NS4A interfered with the IFN production pathway at multiple levels. This is in line with the observation that DENV1 NS4A can inhibit TBK1-induced innate immune signaling (Dalrymple et al., 2015). Moreover, NS4A proteins of several flaviviruses were found to induce autophagy (Blazquez et al., 2014; Liang et al., 2016; McLean et al., 2011; Tan et al., 2023), which was proposed as another mechanism used by flaviviruses to evade the IFN response since the absence of autophagy increases IFN production and signaling in these studies (Hu et al., 2020; Jin et al., 2013). In addition, ZIKV NS4A was recently shown to induce mitophagy, thereby inhibiting MAVS-induced IFN responses (Lee and Shin, 2023). Further in-depth studies on the effects of USUV NS4A on the various host processes is required to fully understand by which additional mechanisms USUV NS4A interferes with the IFN response.

A limitation of our study is that most of our results were obtained by overexpressing proteins and therefore the role of USUV NS4A in inhibiting MAVS signaling in the context of infection remains to be studied. Studies to address this in infected cells, require a good quality antibody against USUV NS4A, and our attempts to generate such an antiserum unfortunately failed. Moreover, our results indicated that NS4A is likely not the only viral protein that counteracts the IFN pathway during infection and it will be complicated to dissect the contributions of the individual proteins. This is further complicated by the fact that many proteins have multiple functions in the viral replication cycle and interactions with the hosts which would complicate mutagenesis studies. It is plausible that several viral proteins can work in concert to inhibit the IFN response. For example, it was shown that DENV NS4B causes elongation of the mitochondria to reduce innate immune signaling (Chatel-Chaix et al., 2016), which could add to the effect of NS4A on MAVS signaling. In support of this, we observed that co-expression of USUV NS4A and NS4B together in the luciferase assay reduced the IFN-β promoter activity to a larger extent than when the proteins were expressed individually (data not shown). It remains to be investigated how these viral proteins might work together to counteract MAVS-dependent signaling.

Our study showed that the NS4A protein from a variety of flaviviruses can inhibit the MAVS mediated interferon response. This implies that this function of NS4A is conserved between flaviviruses, and that it might play an essential role in the replication of flaviviruses in mammalian cells. However, we observed differences in the pulldown efficiencies after the coimmunoprecipitation with MAVS and the extent to which NS4A inhibited the IFN-β reporter. Moreover, TM3 from USUV NS4A was dispensable for its interaction with MAVS, while another study showed that DENV TM3 was required for this interaction (He et al., 2016). This suggests that the mechanism by which NS4A counteracts the MAVS-induced IFN response might differ for the various flaviviruses. It would therefore be of great value to decipher the exact amino acids in NS4A that are involved in the interaction with MAVS, so we can better understand the functional differences between NS4A from the various flaviviruses. Moreover, this knowledge could aid in creating mutant viruses that are defective in interfering with MAVS signaling, and might be attenuated. In summary, our study provided more insight into evasion of the innate immune response by USUV and enhanced our understanding of the role of flavivirus NS4A in general, which is important for better understanding the role of NS4A in flavivirus pathogenesis, and could contribute to rational design of live-attenuated vaccines.

## Funding

This publication is part of the project ‘Preparing for Vector-Borne Virus Outbreaks in a Changing World: a One Health Approach’ (NWA.1160.1S.210), which is (partly) financed by the Dutch Research Council (NWO). This project has received funding from the European Union's Horizon 2020 research and innovation program under grant agreement No 952373.

## CRediT authorship contribution statement

**Tessa Nelemans:** Writing – original draft, Visualization, Methodology, Investigation, Formal analysis, Conceptualization. **Ali Tas:** Methodology, Investigation. **Marjolein Kikkert:** Writing – review & editing, Supervision, Methodology, Funding acquisition, Conceptualization. **Martijn J. van Hemert:** Conceptualization, Methodology, Writing – review & editing, Supervision, Funding acquisition.

## Declaration of competing interest

The authors declare that they have no known competing financial interests or personal relationships that could have appeared to influence the work reported in this paper.

## Data Availability

Data will be made available on request.

## Appendix Supplementary materials

Image, application 1

Image, image 2

Image, application 3
