# Supplementary material for: Usutu virus NS4A suppresses the host interferon response by disrupting MAVS signaling
Source: Virus Res. 2024 Jul 9;347:199431. doi: 10.1016/j.virusres.2024.199431 (PMC11292556; doi:10.1016/j.virusres.2024.199431)

Fig. S1. USUV induces a delayed, strong IFN response in A549 cells. (A-D) A549 cells were infected with USUV (MOI = 1) or SeV (100 HAU/mL) and lysates were collected at the indicated time points. Total RNA was isolated and IFN-β (A), IFIT2 (B) and ISG15 (C) mRNA levels were measured by RT-qPCR. Gene expression was normalized to RPL13a and the fold change versus mock-infected cells calculated. The relative amount of viral genome copies were calculated as fold change relative to the levels at 2 hpi (D). Graphs show a representative example of three independent experiments. (E) A549 cells were transfected with poly(I:C) (1μg/mL) and lysates were collected at 6 hpt. Total RNA was isolated and IFN-β, IFIT2 and ISG15 mRNA levels were measured by RT-qPCR. Gene expression was normalized to RPL13a and the fold change versus mock-transfected cells calculated. Means ± standard deviation of two independent experiments are shown.


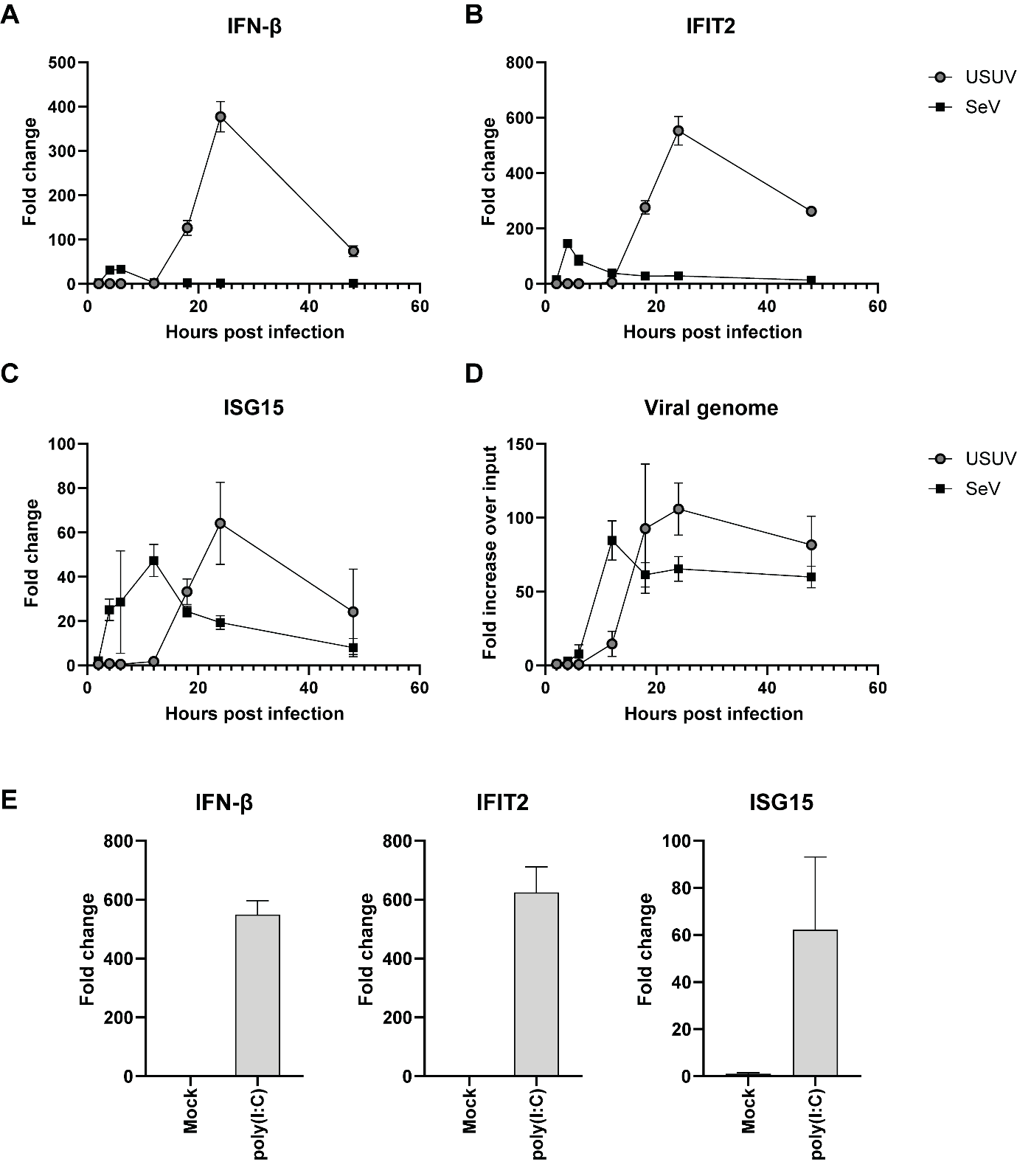

Supplement: Supplementary file 1 [file mmc1.docx]
