# Supplementary figures and images for: Usutu virus NS4A suppresses the host interferon response by disrupting MAVS signaling
Source: Virus Res. 2024 Jul 9;347:199431. doi: 10.1016/j.virusres.2024.199431 (PMC11292556; doi:10.1016/j.virusres.2024.199431)

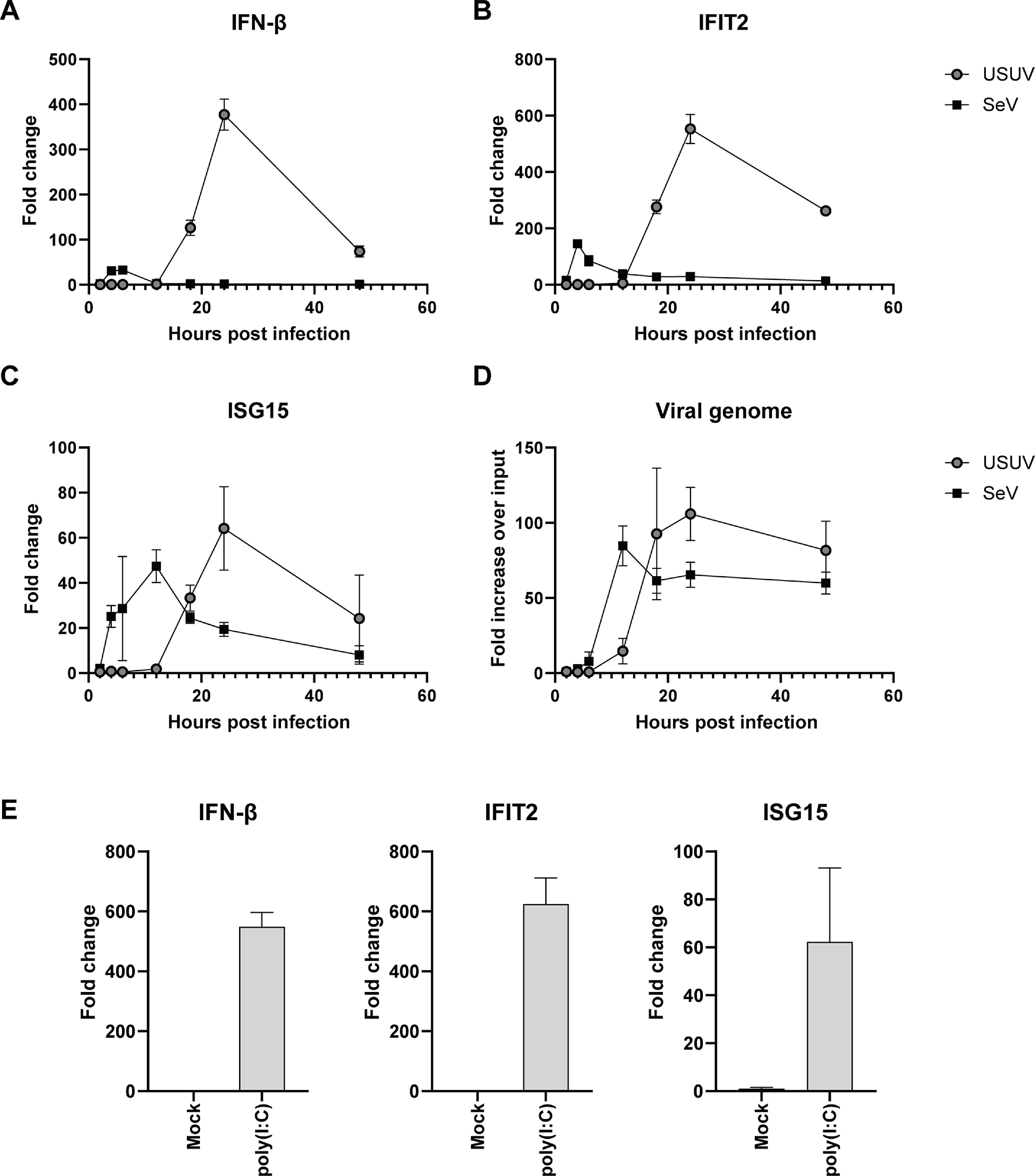

Supplement: Supplementary file 2 [file mmc2.jpg]
