# Supplementary material for: Usutu virus NS4A suppresses the host interferon response by disrupting MAVS signaling
Source: Virus Res. 2024 Jul 9;347:199431. doi: 10.1016/j.virusres.2024.199431 (PMC11292556; doi:10.1016/j.virusres.2024.199431)
Supplement: Supplementary file 3 [file mmc3.docx]

# **Supporting information**

**Table S1** Primers for plasmid construction

| **Primer name** | **Primer sequence** **(5’-3’)** |
| --- | --- |
| USUV-NS1-F | TAAGCACCCGGGATGGATCGATCCATCGCACTGG |
| USUV-NS1-R | CGCGCGGCTAGCTTAGGTGCTGTCCAGGCCCAGCAGAGGATTGGGGATGGGCTTCCCGGCACTGACGCTGGATTTTAC |
| USUV-NS1’-F1 | TTGGCAAAGAATTCGAGCTCACGCGTATGGATCGATCCATCGCACTGG |
| USUV-NS1’-R1 | CTG**A**AAAAGGATCAATCATGTCACTCC* |
| USUV-NS1’-F1 | CATGATTGATCCTTTT**T**CAGTTGGGCCTTCTGGTGATG* |
| USUV-NS1’-R2 | GAGGATTGGGGATGGGCTTCCCGTGTAAGTAATTCCCCCAAG |
| USUV-NS2A-F | TAAGCACCCGGGATGTACCGGAGTGACATGATTG |
| USUV-NS2A-R | GCGCGCCTCGAGTTAGGTGCTGTCCAGGCCCAGCAGAGGATTGGGGATGGGCTTCCCCCGCTTTTTGTTGGGATTG |
| USUV-NS2B3-F | TAAGCACCCGGGATGGGCTACCCCTACGACGTGCCAGATTACGCTGGATGGCCGGCTACAGAAG |
| USUV-NS2B3-R | GCGCGCCTCGAGTTAGGTGCTGTCCAGGCCCAGCAGAGGATTGGGGATGGGCTTCCCTCGCTTACCAGCCGCAAA |
| USUV-NS4A-F | TAAGCACCCGGGATGGGCTACCCCTACGACGTGCCAGATTACGCTTCAGCTGTGGGATTCCTTG |
| USUV-NS4A-R | GCGCGCCTCGAGTTATCGTTGCTTCTCAGGTTCAG |
| USUV-NS4B-F | TAAGCACCCGGGATGTCCCAGACAGACAACCAG |
| USUV-NS4B-R | GCGCGCCTCGAGTTAGGTGCTGTCCAGGCCCAGCAGAGGATTGGGGATGGGCTTCCCTCGTTTGCAGGCCGGTTTG |
| USUV-NS5-F | TAAGCACCCGGGATGGGGAAGCCCATCCCCAATCCTCTGCTGGGCCTGGACAGCACCGGAAGACCAGGAGGAAGGAC |
| USUV-NS5-R | GCGCGCCTCGAGTTACAAAACCCTGTCCTCTTGG |
| WNV-NS4A-F | TAAGCACCCGGGATGGGCTACCCCTACGACGTGCCAGATTACGCTTCACAAATCGGGCTCGTTG |
| WNV-NS4A-R | CGCGCGCTCGAGTTAGCGCTGCTTTTCCGGCTCC |
| ZIKV-NS4A-F | TAAGCACCCGGGATGGGCTACCCCTACGACGTGCCAGATTACGCTGGAGCGGCTTTTGGAGTG |
| ZIKV-NS4A-R | CGCGCGCTCGAGTTATCTTTGCTTTTCTGGCTCAGG |
| MAVS(1-513)-F | TATGACTCTAGAGCCACCATGGACTACAAGGACGACGATG |
| MAVS(1-513)-R | TCGTTAAAGCTTCTAAGGTGAGGGCCTGTGGCATGG |
| MAVS(1-173)-F | TAAGCACCCGGGATGGACTACAAGGACGACGATGACAAGATGCCGTTTGCTGAAGAC |
| MAVS(1-173)-R | TGCTTACTCGAGCTATGGATTCCTTGGGATGGC |
| MAVS(78-173)-F | TAAGCACCCGGGATGGACTACAAGGACGACGATGACAAGGGCTGTGAGCTAGTTGATC |
| MAVS(78-173)-R | TGCTTACTCGAGCTATGGATTCCTTGGGATGGC |
| MAVS(78-540)-F | TAAGCACCCGGGATGGACTACAAGGACGACGATGACAAGGGCTGTGAGCTAGTTGATC |
| MAVS(78-540)-R | TGCTTACTCGAGCTAGTGCAGACG |
| MAVS(174-540)-F | TAAGCACCCGGGATGGACTACAAGGACGACGATGACAAGGATGGTGGCCCCCTGGA |
| MAVS(174-540)-R | TGCTTACTCGAGCTAGTGCAGACG |
| EV-pCAGGS-GFP-F | GGGGGAGCTCATGGTGAGCAAGGGCGAGGAGC |
| EV-pCAGGS-GFP-R | GGGCCCTTTCTCGAGGGGCCCTTTGCATGCTTATTACTTGTACAGCTCGTCCATGCCGTG |
| USUV-NS4A-2K-F | TAAGCACCCGGGATGGGCTACCCCTACGACGTGCCAGATTACGCTTCAGCTGTGGGATTCCTTG |
| USUV-NS4A-2K-R | CGCGCGCTCGAGTTAGGCAGCCACCACTCCCAC |
| USUV-NS4A-ΔTM3-F | TAAGCACCCGGGATGGGCTACCCCTACGACGTGCCAGATTACGCTTCAGCTGTGGGATTCCTTG |
| USUV-NS4A-ΔTM3-R | GCGCGCCTCGAGTTAGACGTCAGCCATCCACAGA |
| USUV-NS4A-ΔTM2-3-F | TAAGCACCCGGGATGGGCTACCCCTACGACGTGCCAGATTACGCTTCAGCTGTGGGATTCCTTG |
| USUV-NS4A-ΔTM2-3-R | GCGCGCCTCGAGTTACTGAACGAGGAGCAAGAAAAC |
| USUV-NS4A-ΔTM1-3-F | TAAGCACCCGGGATGGGCTACCCCTACGACGTGCCAGATTACGCTTCAGCTGTGGGATTCCTTG |
| USUV-NS4A-ΔTM1-3-R | GCGCGCCTCGAGTTACGGCAACTCTTCAAGGGC |
| USUV-NS4A-TM1-3-F | TAAGCACCCGGGATGGGCTACCCCTACGACGTGCCAGATTACGCTGATGCTCTTGAGACTATAACAC |
| USUV-NS4A-TM1-3-R | GCGCGCCTCGAGTTATCGTTGCTTCTCAGGTTCAG |

*In bold is the nucleotide that was inserted in the sequence to mimic the -1 ribosomal frameshift which results in the production of the NS1’ protein.

**Table S2** RT-qPCR primers for cellular genes and viral genomes

| **Target** | **Forward primer** **(5’-3’)** | **Reverse primer (5’-3’)** |
| --- | --- | --- |
| IFN-β | TGCTCCAGAACATCTTTG | GATGGTTTATCTGATGATAGAC |
| IFIT2 | GGACCAAAGTCTAAATAGGG | GGCACTTGAATTCACATTG |
| ISG15 | TCCTGGTGAGGAATAACAAGGG | GTCAGCCAGAACAGGTCGTC |
| RPL13a | AAGGTGGTGGTCGTACGCTGTG | CGGGAAGGGTTGGTGTTCATCC |
| USUV | TCAGAAAAGACGTGCCAGAG | AAAGTCCTTCCGTCCTTCATG |
| SeV | CAGAGGAGCACAGTCTCAGTGTTC | TCTCTGAGAGTGCTGCTTATCTGTGT |
